# Thinking on your feet: Beauty and auto small businesses maneuver the risks of the COVID-19 pandemic

**DOI:** 10.3389/fpubh.2022.921704

**Published:** 2022-08-16

**Authors:** Denise Moreno Ramírez, Shannon Gutenkunst, Jenna Honan, Maia Ingram, Carolina Quijada, Marvin Chaires, Sam J. Sneed, Flor Sandoval, Rachel Spitz, Scott Carvajal, Dean Billheimer, Ann Marie Wolf, Paloma I. Beamer

**Affiliations:** ^1^Community, Environment and Policy Department, Mel and Enid Zuckerman College of Public Health, The University of Arizona, Tucson, AZ, United States; ^2^BIO5 Institute, The University of Arizona, Tucson, AZ, United States; ^3^Health Promotion Sciences Department, Mel and Enid Zuckerman College of Public Health, The University of Arizona, Tucson, AZ, United States; ^4^Sonora Environmental Research Institute, Tucson, AZ, United States; ^5^Epidemiology and Biostatistics Department, Mel and Enid Zuckerman College of Public Health, The University of Arizona, Tucson, AZ, United States

**Keywords:** occupational health, COVID-19 pandemic, chemical exposures, small businesses, vaccination, disinfection, Arizona, health equity

## Abstract

On March 11, 2020, the World Health Organization officially declared SARS-CoV-2 a pandemic, and governments and health institutions enacted various public health measures to decrease its transmission rate. The COVID-19 pandemic made occupational health disparities for small businesses more visible and created an unprecedented financial burden, particularly for those located in communities of color. In part, communities of color experienced disproportionate mortality and morbidity rates from COVID-19 due to their increased exposure. The COVID-19 pandemic has prompted the public to reflect on risks daily. Risk perception is a critical factor influencing how risk gets communicated and perceived by individuals, groups, and communities. This study explores competing risk perceptions regarding COVID-19, economic impacts, vaccination, and disinfectant exposures of workers at beauty salons and auto shops in Tucson, Arizona, using a perceived risk score measured on a scale of 1–10, with higher scores indicating more perceived risk. The primary differences between respondents at beauty salons and auto shops regarding their perceived risks of COVID-19 vaccination were between the vaccinated and unvaccinated. For every group except the unvaccinated, the perceived risk score of getting the COVID-19 vaccine was low, and the score of not getting the COVID-19 vaccine was high. Study participants in different demographic groups ranked economic risk the highest compared to the other five categories: getting the COVID-19 vaccine, not getting the COVID-19 vaccine, COVID-19, disinfection, and general. A meaningful increase of four points in the perceived risk score of not getting the COVID-19 vaccine was associated with a 227% (95% CI: 27%, 740%) increase in the odds of being vaccinated. Analyzing these data collected during the coronavirus pandemic may provide insight into how to promote the health-protective behavior of high-risk workers and employers in the service sector during times of new novel threats (such as a future pandemic or crisis) and how they process competing risks.

## Introduction

“There is a big difference between those who take risks and those who are victimized by risks others take.” —Ulrich Beck (*Risk Society*, 1986).

On March 11, 2020, the World Health Organization officially declared SARS-CoV-2 a pandemic (1). By August 2020, the spread of the virus had resulted in 20 million cases and 700,000 deaths worldwide (1). Governments and health institutions enacted various public health measures to decrease the rate of transmission. In the United States (U.S.), this resulted in business shutdowns due to social distancing and shelter-in-place guidelines (2–4). Individuals in many service industries are frontline workers because they physically report to work (often working within six feet of others) and are more likely to be exposed to the COVID-19 disease whether they are officially defined as such (5).

Previous studies suggest the service industry is more likely to employ workers of low socioeconomic status and people of color (6, 7). Occupational health disparities attributable to contaminant exposures in these work environments have led to the overrepresentation of historically neglected populations in this sector. Owners and employees often lack access to resources and monetary funds to implement recommended costly interventions (e.g., industrial hygiene consultants, ventilation systems) (8, 9). The COVID-19 pandemic added new burdens as business owners and employees had to use their already limited resources to purchase additional equipment and products to protect themselves and their clients (e.g., surgical masks, disinfection products, air purifiers) while working in person and many times near others, even when recommendations from government agencies were constantly changing. Trust in government and public health organizations influence an individual's willingness to be vaccinated and use other interventions (10). At the initiation of the pandemic, officials were scrambling to establish facts about this novel virus. Thus risk communication was impacted as it changed constantly and often contradicted public health messaging. Initial risk messaging from government leaders contributed to the confusion because the pandemic took on a political association and was less based on fast-changing facts (10, 11). These workers relied on their knowledge and self-efficacy to navigate and interpret competing risks.

The COVID-19 pandemic made occupational health disparities for small businesses more visible and created an unprecedented financial burden, particularly for those located in communities of color (2). Significant impacts on the supply chain worldwide, plus border closures, added to market fluctuations and economic impacts (12). In this study, we define small businesses as those with <100 employees and have even focused on microbusinesses with much fewer employees. Small businesses are considered important economic drivers in the U.S. (13). Before the pandemic, small business owners found themselves in a precarious financial situation, with this event further exposing the disproportionate effects on economics and health of these businesses (14). Small businesses have had to weigh the constantly changing risks of workplace SARS-CoV-2 transmission against financial burdens and social costs caused by business closure or reduced number of staff and clients. The use of face masks, changes to disinfection practices, vaccine requirements, and more are the responsibility of business owners and workers.

While acknowledging structural conditions that manifest in health disparities, understanding risk perception is one approach to help determine health-protective behaviors that could mitigate health effects (15). Douglas and Wildavsky (16) propose a cultural risk perception model suggesting risk is a “social process,” emphasizing that risk cannot be calculated with precision. A cultural approach to risk can highlight how a community relates “natural dangers to moral defects” (16). The key is to determine what characteristics of “social life” result in an individual's “typical risk portfolio.” In other words, the social structure we individually belong to strongly contributes to the risks we are willing to accept. “To alter risk selection and risk perception, then, would depend on changing the social organization” (16). Because a universal concept of risk that encompasses the “social life” of everyone is nonexistent, there is also no singular interpretation. Yet, characteristics that may influence risk perception can include knowledge, personality, economics, politics, and culture (17).

Subjective risk describes a person's perceived chance that something harmful will happen. A personal assessment of their vulnerability to the threat is not based on a mathematical formula characterizing the type of risk (18, 19). A person's perspective may make some risks appear more alarming than others. Subjective risk is higher in an individual if involuntary, catastrophic, unequal, unfamiliar, or complex (20). Usually, decisions about risk are less influenced by information regarding the risk itself (21). Therefore, the risk perception of an emerging hazard may be more emotionally-based (11). Outrage (response) and the nature of the hazard (number of people exposed, infected, ill), as well as cultural and economic factors, determine risk perception and response to public health messages such as that of COVID-19 (11). For example, in the COVID-19 pandemic, masks evolved into a political issue even though they are an effective intervention against airborne viruses. The politicization of face masks resulted in their varying use throughout the U.S.

The COVID-19 pandemic has prompted small businesses to reflect on risks daily. In a systematic review, “educational initiatives, proper communication, and timely information” at the community levels were found to promote the successful implementation of public health strategies and decrease misinformation (22). Yet, there is a limited capacity from workplace organizations like the Occupational Safety and Health Administration or the Small Business Administration to help guide many extant businesses. During the pandemic, business owners simultaneously had to keep their employees healthy and their businesses profitable while increasing clientele confidence about safety from COVID-19 transmission. The latter was particularly difficult for industries that did not have viable options for transitioning away from in-person services within 1.83 meters, like beauty salons. Clear and consistent guidance from local, state, and federal authorities, including governments and health organizations, are needed to direct these industries better (23). The COVID-19 vaccine was an added competing interest for individuals working in these businesses. Understanding personal hesitancy toward the vaccine and other interventions is critical to limiting the spread of the disease and mutations and protecting frontline workers.

Risk perception is a critical factor influencing how risk gets communicated by individuals, groups, and communities. It is also a positive driver of the public's acceptance of official measures and recommendations (10, 11). For example, an Italian study concluded that willingness to get vaccinated was shaped by various factors with risk perception being of importance (24). As risk perception increased in participants, so did their willingness to get vaccinated (24). This study explores competing risk perceptions regarding COVID-19, economic impacts, vaccination, and disinfectant exposures of workers at beauty salons and auto shops in Tucson, Arizona, as part of a more extensive study to reduce workplace environmental exposures. A cross-sectional survey was developed and implemented to understand the small business impacts associated with the COVID-19 pandemic.

## Materials and methods

In 2017, researchers established the principal study to understand if applying an industrial-hygiene enhanced community health worker (CHW) intervention can decrease exposures to volatile organic compounds (VOCs) routinely found in beauty and auto small businesses. Individuals from the University of Arizona Mel and Enid Zuckerman College of Public Health (MEZCOPH), Sonoran Environmental Research Institute, Inc. (SERI), and El Rio Health collaborated. With the onset of the COVID-19 pandemic, the study expanded its focus from measuring air concentrations of VOCs to understanding the impacts of the pandemic on the study's population. Activities shifted to designing resources about the novel COVID-19 virus and guidance, with the benefit of maintaining communication and providing support during a demanding period for these small businesses. A cross-sectional survey was developed and implemented as part of this ongoing community-engaged research to understand how the pandemic was impacting businesses and changing their work practices. The survey also included a section to assess the competing risk perceptions regarding viral transmissions, financial hardships, vaccination status, and disinfection exposures. It provides a novel perspective on the impacts of COVID-19 on small businesses in Tucson. It was also an opportunity to better understand the perceptions of workers/managers and employees from small businesses following a catastrophic event that may have influenced workplace decision-making processes.

### Study population and recruitment

Race and ethnicity are socially constructed categories that have real-life implications regarding health disparities (e.g., chronic disease, premature death) (25). Health inequities in the U.S. have been brought to the surface during the COVID-19 pandemic, with Tucson, Arizona being no exception. The Tucson zip codes that are part of the study area contain high poverty rates, urban stress, and lower education attainment (26). They also include the Mexican and Mexican American neighborhoods that are Spanish-speaking in the city. The study area also has among the highest rates of COVID-19 cases and death in the region. This area is already at an increased risk for VOC exposure, and the pandemic may increase chemicals in workplace air due to increased disinfection.

The study targeted small business beauty salons and auto shop owners, managers, and employees in Tucson who were at least 18 years old and spoke either Spanish or English. Recruitment of participants was *via* social media, phone calls, mailed flyers, and poster advertisements. Contact information for the businesses was compiled based on internet searches, social media presence, and driving through targeted neighborhoods. Honan et al. provide detailed recruitment methods (23).

Workers in beauty and auto small businesses responded to the survey between June 8, 2021, and January 25, 2022. The questions were either self-administered online or over the phone to owners, managers, and workers of auto repair shops and beauty salons in Tucson, Arizona. This survey also collected demographic information. A total of 67 individuals representing owners, managers, and employees participated. Individuals who completed the survey received a $25 gift card as compensation for their time and effort.

#### Survey

The development of the COVID-19 small business survey has been described previously (23). An interdisciplinary team from the University of Arizona and SERI designed the survey instrument. This questionnaire captured the perceived risks of the novel COVID-19, COVID-19 vaccination, disinfection activities, economic impacts, and non-occupational health hazards.

The COVID-19 small business risk perception section of the survey was adapted from a previous study (24). Respondents evaluated the risk of activities posed from their perspective. They rated activities using a 10-point Likert scale, with one being something they consider very low risk and 10 being something they consider extremely risky. Table 1 displays individual survey items about perceived risk of specific activities, grouped into broader risk categories. The survey was designed to be succinct and typically completed in 30 min. The survey was submitted to and approved by the University of Arizona Human Subject Protection Program.

**Table 1:** Survey respondents were asked to rate the following specific activities from 1 = very low risk to 10 = extremely risky; individual activities have been grouped into broader risk categories for assessment of these more general categories of risk.

**Broad risk categories**	**COVID-19 vaccination risk**	**COVID-19 risk**	**Disinfection risk**	**Economic risk**	**General risk**
Specific activities	Getting the COVID vaccine	Eating a meal indoors with people who don't live in my home	Using alcohol to disinfect surfaces	Betting a day's income at the casino	Driving a car
	Not getting the COVID vaccine	Eating lunch with coworkers at work—indoors	Using Clorox^®^ wipes to disinfect surfaces	Investing 10% of my annual income in a new business	Drinking and driving
		Eating lunch with coworkers at work—outdoors	Using liquid bleach to disinfect surfaces	Quitting my job or shutting down my business	Firing a gun
		Spending time with family or friends without a face mask	Using Lysol^®^ to disinfect surfaces	Continuing to reduce my work hours or the open hours of my business	Riding a motorcycle
		Being at the grocery store without a face mask	Using Pine-sol^®^ to disinfect surfaces		Listening to loud music
		Being at work without a face mask	Using disinfectant sprays in my workspace		Riding in a car without a seatbelt
			Using disinfectant wipes in		Smoking
			my workspace		Playing soccer
					Exposure to pesticides
					Using Raid^®^

### Data analysis

Survey responses were de-identified before data analysis. The survey data was downloaded from the REDCap (Research Electronic Data Capture) platform available through the University of Arizona Health Sciences. Data were read into the R statistical computing software Mac version 4.1.2 (25) R Core Team Vienna, Austria), cleaned and combined. Additional R packages used included the R tidyverse package for manipulating data (26) and the DescTools package to calculate multiplicity-adjusted *p*-values using Dunnett's test (27). As part of the data cleaning process, data from two participants who had selected “other type of shop” with the text “Automotive headlight restoration and light-duty mechanical work” and “RV and boat repair” were changed to auto shops. Any questionnaires not filled out by someone working at an auto shop or beauty salon or where the participant did not complete the survey were removed.

We tested how perceptions about risk vary across different groups using a longitudinal survey's baseline cross-sectional survey data. First, we tested if vaccination status was related to various demographic variables using Pearson's chi-squared test for categorical variables and linear model analysis of variance (ANOVA) for continuous variables. Next, for each respondent, their average perceived risk score was calculated for the following categories: general risk (average of 10 statements), economic risk (average of four statements), disinfection risk (average of seven statements), COVID-19 risk (average of six statements) (Table 1). We then compared the risk perception of COVID-19 to other activities; Dunnett's test was performed to identify which activities had statistically significantly different mean risk scores compared to COVID-19 risk (28). Additionally, average perceived risk scores for categories of risk, along with perceived risk scores of getting and not getting the COVID-19 vaccine, were ranked for the following demographic groups: vaccinated/not; auto/beauty; employee/manager or owner; Hispanic/not; and Hispanic female, Hispanic male, non-Hispanic female, non-Hispanic male. Finally, we ran a logistic regression model of vaccination status on the perceived risk score of not getting the COVID-19 vaccine, gender, ethnicity, and the gender by ethnicity interaction. An α level of 0.05 was used for all tests of statistical significance.

## Results

Table 2 displays the descriptive statistics of demographic information obtained from cross-sectional survey responses from June 8, 2021, through January 25, 2022 by vaccine status. Sixty-four cross-sectional surveys were analyzed (46.8% employees, 17.7% managers, and 35.5% owners). Originally 67 individuals participated, with three declining to state their vaccination status, and therefore were excluded from the analysis. A mean of six employees worked at each auto shop or beauty salon. Most workers at auto shops were male (72.4%), while most workers at beauty salons were female (86.8%). Approximately half of the workers were of Hispanic ethnicity (53.1%), and a little less than half were non-Hispanic (46.9%). Workers were also White (81.0%), Black or African American (6.9%), more than one race (6.9%), Indigenous/American Indian/Alaskan Native (3.4%), and Asian (1.7%). For the most part, individuals completed trade school (26.6%) or some college (28.1%). Most individuals (82.8%) believed they had enough work and were not currently looking for more or different employment opportunities. The respondents had a mean age of 41 years (SD = 14, range: 21–71).

**Table 2:** Descriptive statistics of demographics by vaccination status for our cross-sectional survey of 64 individuals (survey of 67 individuals, three of whom declined to state their vaccination status) between June 8, 2021 and January 25, 2022 who worked at beauty salons and auto shops in Tucson, AZ, were at least 18 years old, and spoke either Spanish or English.

	**Vaccinated**	**Not vaccinated**	**Total**	***p*** **value**
	**(*N* = 53)**	**(*N* = 11)**	**(*N* = 64)**	
**Shop type**				
Auto	23 (43.4%)	6 (54.5%)	29 (45.3%)	0.50^a^
Beauty	30 (56.6%)	5 (45.5%)	35 (54.7%)	
**Gender**				
Female	34 (64.2%)	4 (36.4%)	38 (59.4%)	0.09^a^
Male	19 (35.8%)	7 (63.6%)	26 (40.6%)	
**Ethnicity**				
Hispanic	30 (56.6%)	4 (36.4%)	34 (53.1%)	0.22^a^
Not Hispanic	23 (43.4%)	7 (63.6%)	30 (46.9%)	
**Race**				
Indigenous/American Indian/Alaska Native	2 (4.2%)	0 (0.0%)	2 (3.4%)	0.78^a^
Asian	1 (2.1%)	0 (0.0%)	1 (1.7%)	
Black or African American	3 (6.2%)	1 (10.0%)	4 (6.9%)	
White	38 (79.2%)	9 (90.0%)	47 (81.0%)	
More than one race	4 (8.3%)	0 (0.0%)	4 (6.9%)	
**Education**				
Some high school	1 (1.9%)	0 (0.0%)	1 (1.6%)	0.73^a^
Completed high school	16 (30.2%)	1 (9.1%)	17 (26.6%)	
Some trade school	1 (1.9%)	0 (0.0%)	1 (1.6%)	
Completed trade school	13 (24.5%)	4 (36.4%)	17 (26.6%)	
Some college	14 (26.4%)	4 (36.4%)	18 (28.1%)	
Completed college or graduate school	8 (15.1%)	2 (18.2%)	10 (15.6%)	
**Working as much as would like?**				
Yes (have enough work)	39 (81.2%)	9 (90.0%)	48 (82.8%)	0.51^a^
No (looking for more work)	9 (18.8%)	1 (10.0%)	10 (17.2%)	
**Employee type**				
Employee	25 (49.0%)	4 (36.4%)	29 (46.8%)	0.61^a^
Manager	8 (15.7%)	3 (27.3%)	11 (17.7%)	
Owner	18 (35.3%)	4 (36.4%)	22 (35.5%)	
**Number employed at shop**				
Mean	6.3	4.1	5.9	0.28^b^
Standard deviation	6.4	2.0	5.9	
Range	0.0–35.0	1.0–8.0	0.0–35.0	
**Age (years)**				
Mean	42.4	37.1	41.4	0.29^b^
Standard deviation	15.1	5.7	14.0	
Range	21.0–71.0	30.0–47.0	21.0–71.0	

a *Pearson's Chi-squared test*.

b *Linear Model ANOVA*.

These small business respondents had high vaccination rates, with 83% reporting they had at least one dose of the COVID-19 vaccine. Gender was the demographic characteristic most strongly associated with vaccination status (*p*-value = 0.09), with women more likely to be vaccinated than men (Table 2). Ethnicity was the next most strongly associated with it (*p*-value = 0.22), with Hispanics being slightly more likely to be vaccinated than non-Hispanics. However, none of these differences were statistically significant at the level of α = 0.05.

Table 3 summarizes the perceived risk score of COVID-19 relative to general risk categories as well as specific activities. Participants regarded activities related to exposure to COVID-19 as a moderate risk, similarly risky to driving a car or firing a gun. Activities perceived as significantly less risky than COVID-19 were disinfection, getting the COVID-19 vaccine, and playing soccer (all with *p* < 0.001) (Table 3). Activities perceived as significantly riskier than COVID-19 included riding a motorcycle (*p* = 0.032), not getting the COVID-19 vaccine (*p* = 0.009), smoking cigarettes, riding in a car without a seatbelt, and drinking and driving (all with *p* < 0.001). Economic risks have a similar mean perceived risk score as exposure to COVID-19 (*p* = 0.099).

**Table 3:** Summary of perceived risk score of COVID (average of six statements) relative to other activities on a Likert scale from 1 (very low risk) to 10 (extremely risky).

**Activity**	**Mean**	**SD**	^*^Mean **difference**	***p*** **value**
General Categories	5.5	2.3		
COVID-19 (average of six statements)				
Disinfection (average of seven statements)	3.2	2.2	−2.2	<0.001
Economic (average of four statements)	6.7	1.8	1.2	0.099
Specific activities	3.3	2.9	−2.2	<0.001
Getting the COVID-19 vaccine				
Playing soccer	3.4	2.4	−2.0	<0.001
Listening to loud music	4.7	2.5	−0.8	0.629
Using Raid	4.9	2.9	−0.6	0.903
Driving a car	5.0	2.5	−0.5	0.966
Firing a gun	5.9	3.2	0.5	0.974
Exposure to pesticides	6.7	2.9	1.3	0.077
Riding a motorcycle	6.9	3.1	1.4	0.032
Not getting the COVID-19 vaccine	7.1	3.4	1.6	0.009
Smoking	7.9	2.7	2.4	<0.001
Riding in a car without a seatbelt	8.3	2.5	2.9	<0.001
Drinking and driving	8.7	2.7	3.2	<0.001

* *The significance of the mean difference of score for each activity compared to that of the reference activity of COVID-19 (average of six statements) was assessed using Dunnett's test, which accounts for these many-to-one comparisons*.

The most significant differences in relative perceived risk rankings (1–6; where one is low perceived risk and six is high perceived risk relative to the other categories) based on the mean perceived risk scores are between the vaccinated and unvaccinated (Table 4). For every group except the unvaccinated, the perceived risk score of getting the COVID-19 vaccine was low (1–2), and the score of not getting the COVID-19 vaccine was high (4–6). Respondents with an unknown vaccination status scored like those vaccinated or more conservatively regarding COVID-19 risks. The perceived risk ranking of not getting the COVID-19 vaccine was at the extreme between vaccination status, whereas the ranking of the perceived risk of COVID-19 was generally considered moderate (2–3) and similar between the two. Disinfection risk had low rank (1) for the vaccinated, whereas it had higher rank (4) for the unvaccinated. Generally, regardless of vaccination status, economic risks were scored high (4–6, with the average rank from all survey respondents 5).

**Table 4:** Perceived risk rankings for various demographic groups, based on the mean perceived risk scores presented in Table A.1 in the Appendix. These rankings range from 1–6 for the six categories listed in the six right-most columns in the table, where 1 is low perceived risk and 6 is high perceived risk relative to the other categories.

**Demographic group**	**Getting the COVID-19 vaccine**	**Not getting the COVID-19 vaccine**	**COVID-19**	**Disinfection**	**Economic**	**General**
All	2	6	3	1	5	4
Vaccinated	2	6	3	1	5	4
Not vaccinated	3	1	2	4	6	5
Unknown vacc. status	1	6	5	2	4	3
Auto	2	4	3	1	6	5
Beauty	1	6	3	2	5	4
Employee	2	6	3	1	5	4
Manager or owner	2	4	3	1	6	5
Unknown employee type	1	6	5	2	4	3
Hispanic	1	6	3	2	5	4
Not Hispanic	2	6	3	1	5	4
Hispanic female	1	6	3	2	4	5
Hispanic male	2	6	3	1	5	4
Not Hispanic female	1	6	3	2	5	4
Not Hispanic male	2	4	3	1	6	5

To determine the relative importance of the perceived risk score of not getting the COVID-19 vaccine and demographic variables in association with vaccination status, we ran a logistic regression model of vaccination status on the perceived risk score of not getting the COVID-19 vaccine and the two demographic variables with the strongest associations with vaccination status in this study (gender and ethnicity), along with their interaction. The logistic regression results are shown in Table 5. The perceived risk score of not getting the COVID-19 vaccine was more strongly associated with vaccination status (*p* = 0.01) than either gender, ethnicity, or their interaction, all of which were not statistically significant. A meaningful change in the perceived risk score of not getting the COVID-19 vaccine was four points, because that is the difference in points between the first quartile (score = 5) and median (score = 9) in our sample. A four-point increase in the perceived risk score of not getting the COVID-19 vaccine corresponded to an increase in the odds ratio (OR) of being vaccinated by a factor of 3.27 (95% CI: 1.27, 8.40). As a concrete example from our model, for the lowest vaccinated group in our sample (i.e., non-Hispanic males), a change in the perceived risk score of not getting the COVID-19 vaccine from 5 to 9 corresponded to a change in the probability of being vaccinated from 66% (95% CI: 39%, 86%) to 86% (95% CI: 57%, 97%).

**Table 5:** Logistic regression model results for COVID-19 vaccination status (not vaccinated = 0; vaccinated = 1) on the perceived risk score of not getting the COVID-19 vaccine (range: 1–10), gender (0 = female; 1 = male), ethnicity (0 = Hispanic; 1 = Not Hispanic), and the gender by ethnicity interaction. *N* = 61 (survey of 67 individuals, six observations were omitted because of missing information).

**Term**	**Coefficient Estimate**	SE^*^	***Z*** **statistic**	***p*** **-value**
Intercept	0.78	0.90	0.87	0.39
Perceived risk score of not getting the COVID-19 vaccine	0.30	0.12	2.46	0.01
Gender	−1.10	1.41	−0.78	0.44
Ethnicity	−0.56	1.37	−0.41	0.68
Gender ^*^ Ethnicity	0.07	1.94	0.04	0.97

* *SE = Standard Error*.

The multiplicative increase in the odds ratio (OR) of being vaccinated associated with a Δ-point increase in the perceived risk score of not getting the COVID-19 vaccine is calculated as exp (0.30 ^*^ Δ), where 0.30 is the coefficient estimate given in the table below.

## Discussion

Analyzing data collected during the COVID-19 pandemic may provide insight into how to promote the health-protective behavior of vulnerable workers and employers during times of new novel threats (such as future pandemics or crises) and insight into how such workers and employers process competing risks. In this study, the primary differences between respondents at beauty salons and auto shops regarding their perceived risks of COVID-19 vaccination are between the vaccinated and unvaccinated. Unvaccinated participants' perceived risk of not getting the COVID-19 vaccine was the lowest, suggesting little fear or worry about the disease and higher anxiety and worry about the vaccine. Previous studies showed that the perceived risk of the disease a vaccine protects against is the main factor influencing vaccination status (29–31). The vaccine's safety is also an influencing factor that can help explain racial and ethnic differences in status (31). Lower vaccine acceptability for individuals identifying as non-Latinx African American, of low income, lacking health insurance, and conservative-leaning have been documented (32), which may typify the unvaccinated respondents of this investigation.

In this analysis, the perceived risk of not getting the COVID-19 vaccine is a more meaningful indicator of vaccination status than gender, ethnicity, or other demographic characteristics typically associated with vaccination status. A previous investigation has shown that using only demographic factors limits explanatory models of why individuals are hesitant to get the COVID-19 vaccination, whereas identifying drivers of and barriers to vaccination is a more informative approach (33). An individual's perceived risk is determined by a combination of factors that include technically and socially derived information (34). Sandman originated the concept of outrage, which refers to the public's collective factors when considering if exposure to a hazard (risk) is acceptable (35). Outrage factors that may contribute to respondents' perceived risk of not getting the COVID-19 vaccine may include voluntariness (assume risk is voluntary), control (can prevent or control), fairness (no greater risk than others), and diffusion in time and space (spread over a large population or concentrated) (36). Because a barrier to vaccination success is public hesitancy, a longitudinal understanding of the perceived risks of COVID-19 and vaccines will be crucial to adjusting the scientific communication about the vaccine.

Our results demonstrate that participating workers in small service-industry businesses highly accepted the COVID-19 vaccine (83% of the respondents in this study reported at least receiving one dose). Vaccination rates were between 52 and 80% across Pima County (first dose) during the study period (37). Vaccinated respondents may also feel safer in situations that may expose them to COVID-19, which may explain why the vaccinated and the unvaccinated in this study have similar rankings of COVID-19 risks and thus likely similar levels of fear about the COVID-19 disease. The timing of the cross-sectional study overlaps with when individuals were receiving vaccine second doses and starting booster shots, a period during the pandemic when confidence was high. This public confidence in the first dose vaccine may have influenced the responses of the vaccinated individuals.

Respondents scored the perceived risk of disinfection significantly lower than that of COVID-19. This result could translate to more frequent use of disinfection products to decrease COVID-19 exposure and increase client confidence regarding the safety of visiting a business. The intensification of hygiene efforts could subsequently increase worker exposure to VOCs, as many disinfectants contain these chemicals. Before the pandemic, we measured VOC exposures to be high among workers during cleaning activities in this study. A U.S. EPA study concluded that exposure to airborne pollutants is high when using VOC-containing products, and air concentrations can persist “long after the activity is completed” (38).

Additionally, the health effects of exposure to VOCs vary greatly and can result in asthma and cardiovascular disease, which are risk factors for COVID-19 complications (39). Continued monitoring of these compounds will be essential to ascertain the pandemic's impact on VOC levels in beauty and auto small businesses. The perceived risk ranking of disinfection was moderately risky for unvaccinated respondents but low risk for vaccinated ones (minor difference). A possible explanation for the former might be a wariness of disinfection chemicals. For example, a spike in disinfectant poisonings due to confusion about public messaging resulted in a jump in calls to poison control centers during the pandemic (40). Salvadori and Lauriola concluded in their study that promoting hygiene and cleaning was due to the “negative affective attitude toward the COVID-19 and meditated by an affective appraisal of risk” (15). Interestingly, the vaccinated group did not have a high-risk perception of disinfection, which may indicate a lack of public awareness about the COVID-19-related poisonings or the VOCs contained in the products.

Study participants ranked economic risk among the highest (5/6) among the following six categories: getting the COVID-19 vaccine, not getting the COVID-19 vaccine, COVID-19, disinfection, economic, and general (Table 4). For small businesses, economic impacts during the COVID-19 pandemic were high because of social distancing and shelter-in-place guidelines (2). Also, small businesses had higher financial and health disparities before the onset of the COVID-19 pandemic, making it difficult to respond to and recover from the disruptions associated with COVID-19 (3). Bartik et al. concluded that 43% of the small businesses surveyed were temporarily closed, and employment fell by 40% while dealing with expenses through cuts, debt, or bankruptcy (2). Perceived economic risk is high because of the lived experiences of workers at small businesses and the precarious nature of their business even before the COVID-19 pandemic.

Questions on general risk were incorporated into the cross-sectional survey to understand how individuals ranked these risks compared to COVID-19. General risk was moderate to high between the vaccinated and unvaccinated, respectively. It suggests that respondents' risk perception toward everyday activities may be typical. General risks were scored higher than COVID-19 disinfection and COVID-19 risk.

A limitation of the study is its small sample size, which can impact the statistical power of the analysis. Also, data collected in the survey was self-reported behavior and not actual behavior observed. During data collection, risk perceptions about COVID-19 and associated vaccines may have become more positive as vaccination rates nationwide also improved. This study did not include a survey of individuals before vaccines were widely available. Although vaccines were widely available during the study period, participants experienced waves of COVID-19 variants (Delta, Omicron) that may have also influenced health behavior and COVID-19 risk perception. Additionally, vaccinated individuals may be more likely to respond to the cross-sectional survey, biasing survey results.

On a broader scale, the results of this study add to the growing literature about small business beauty salons and auto shops impacted during the COVID-19 pandemic. These businesses shifted into survival mode, maneuvering economics, interventions, and COVID-19. Understanding the risk perception of these worker populations can also strengthen national efforts to communicate actionable steps during a public health crisis that can help reduce anxiety and fear by increasing the sense of agency of these individuals.

## Conclusion

This study documented workers' risk perceptions from beauty salon and auto shop small businesses located in Tucson, Arizona, during the COVID-19 pandemic. These less visible and disproportionally frontline workers from service industries had higher vaccination rates than the general population. Future messaging targeting these small businesses should focus on vaccine-hesitant individuals to increase their perceived risk of not getting the COVID-19 vaccine (disease saliency). Vaccines are a crucial line of defense for workers considered frontline or essential. Messaging about vaccination should address safety concerns, provide actionable steps, and impact emotion. This is even more important as a Center for Disease Control recent study revealed that only about half of the people eligible for a booster vaccine have not received one (41). For example, because the virus that causes COVID-19 is novel, researchers are still trying to understand the long-term impacts on human health. Communicating the long hauler effects of the COVID-19 disease such as fatigue, cognitive problems, rapid heartbeat, and shortness of breath may help (42).

Additionally, an unintentional increase in exposure to disinfectants is possible during the study period. Understanding these exposures is necessary to build trust and communicate solutions. In the future, it will be essential to continue monitoring workers at small businesses and their associated changes in perception of risk of COVID-19, because the pandemic is not over. Also, expanding the study to other areas that were not as impacted by the pandemic will help determine which disparities increased during this public health crisis and how they directly impacted these industries. Future results on this topic should also be translated to policymakers to make further recommendations to safeguard worker health.

## Data availability statement

The raw data supporting the conclusions of this article will be made available by the authors, without undue reservation in compliance with the study's human subjects protocol.

## Ethics statement

The studies involving human participants were reviewed and approved by University of Arizona Human Subject Protection Program. The patients/participants provided their written informed consent to participate in this study.

## Author contributions

DM interpreted the quantitative data and wrote the original draft of this manuscript. SG analyzed and interpreted the data and contributed to the writing of the manuscript. JH developed the RedCAP to collect survey responses and contributed to the editing and writing of the manuscript. CQ managed the field study, translated study materials, collected survey responses, and contributed to the editing of the manuscript. MC collected survey responses and contributed to the editing of the manuscript. SS contributed to the editing of the manuscript. FS directed engagement for the project and managed recruitment for the study. SC designed the study and contributed to the editing and writing of the manuscript. MI assisted with creating the survey, training on survey administration and contributed to the editing of the manuscript. RS designed the recruitment material. DB designed the quantitative methodology, interpreted the quantitative data, and contributed to the writing of the manuscript. PB conceptualized the study, co-created the survey, interpreted the quantitative data, reviewed the manuscript in detail, and contributed to the writing of the manuscript. All authors contributed to the article and approved the submitted version.

## Funding

This project is support by NIH grants R01 ES028250-03S1and P30 ES006694. DM is supported by T32 ES007091. The publication's contents are solely the responsibility of the authors and do not necessarily represent the official views of the National Institutes of Health.

## Conflict of interest

The authors declare that the research was conducted in the absence of any commercial or financial relationships that could be construed as a potential conflict of interest.

## Publisher's note

All claims expressed in this article are solely those of the authors and do not necessarily represent those of their affiliated organizations, or those of the publisher, the editors and the reviewers. Any product that may be evaluated in this article, or claim that may be made by its manufacturer, is not guaranteed or endorsed by the publisher.

## Author disclaimer

The publication's contents are solely the responsibility of the authors and do not necessarily represent the official views of the National Institutes of Health.
