# Supplementary material for: Thinking on your feet: Beauty and auto small businesses maneuver the risks of the COVID-19 pandemic
Source: Front Public Health. 2022 Aug 16;10:921704. doi: 10.3389/fpubh.2022.921704 (PMC9465998; doi:10.3389/fpubh.2022.921704)
Supplement: Supplementary file 1 [file Table_1.DOCX]

Supplementary Material

# Supplementary Tables

| Table A.1 Mean perceived risk scores and standard deviations (SD) for various demographic groups. | | | | | | | | | | | | | |
| --- | --- | --- | --- | --- | --- | --- | --- | --- | --- | --- | --- | --- | --- |
|  | | **Getting the COVID-19 vaccine** | | **Not getting the COVID-19 vaccine** | | **COVID-19** | | **Disinfection** | | **Economic** | | **General** | |
| **Demographic group** | **N** | **Mean** | **SD** | **Mean** | **SD** | **Mean** | **SD** | **Mean** | **SD** | **Mean** | **SD** | **Mean** | **SD** |
| All | 67 | 3.3 | 2.9 | 7.1 | 3.4 | 5.5 | 2.3 | 3.2 | 2.2 | 6.7 | 1.8 | 6.3 | 1.6 |
| Vaccinated | 53 | 3.2 | 2.8 | 7.6 | 3.2 | 5.6 | 2.2 | 2.9 | 1.9 | 6.7 | 1.7 | 6.4 | 1.5 |
| Not vaccinated | 11 | 4.3 | 3.6 | 3.9 | 3.0 | 4.1 | 2.2 | 4.3 | 2.9 | 6.5 | 2.1 | 5.7 | 1.9 |
| Unknown vaccination status | 3 | 2.0 | 1.0 | 9.3 | 1.2 | 7.7 | 2.2 | 5.8 | 3.7 | 6.6 | 2.0 | 5.9 | 1.6 |
| Auto | 29 | 3.5 | 3.1 | 5.9 | 3.7 | 4.5 | 2.2 | 2.8 | 1.6 | 6.5 | 1.7 | 6.0 | 1.8 |
| Beauty | 38 | 3.1 | 2.8 | 8.0 | 2.8 | 6.2 | 2.1 | 3.6 | 2.6 | 6.9 | 1.8 | 6.5 | 1.4 |
| Employee | 30 | 3.8 | 3.2 | 7.6 | 3.2 | 5.7 | 2.3 | 3.4 | 2.2 | 6.6 | 1.9 | 6.0 | 1.6 |
| Manager or owner | 34 | 3.0 | 2.7 | 6.5 | 3.7 | 5.1 | 2.1 | 3.0 | 2.0 | 6.8 | 1.7 | 6.6 | 1.6 |
| Unknown employee type | 3 | 1.7 | 0.6 | 8.3 | 0.6 | 7.2 | 3.6 | 4.5 | 4.8 | 6.8 | 2.2 | 5.9 | 1.6 |
| Hispanic | 34 | 3.2 | 3.0 | 7.3 | 3.4 | 5.7 | 2.2 | 3.4 | 2.2 | 6.7 | 1.9 | 6.5 | 1.6 |
| Not Hispanic | 33 | 3.3 | 2.8 | 6.9 | 3.5 | 5.3 | 2.4 | 3.1 | 2.2 | 6.7 | 1.6 | 6.0 | 1.6 |
| Hispanic female | 26 | 3.3 | 3.0 | 6.9 | 3.6 | 5.7 | 2.3 | 3.5 | 2.5 | 6.8 | 2.1 | 6.8 | 1.7 |
| Hispanic male | 8 | 3.1 | 2.9 | 8.2 | 2.4 | 5.6 | 2.2 | 2.9 | 1.4 | 6.4 | 1.6 | 5.8 | 1.3 |
| Not Hispanic female | 15 | 2.9 | 1.8 | 8.3 | 2.2 | 6.3 | 2.1 | 3.4 | 2.4 | 7.5 | 1.1 | 6.3 | 0.9 |
| Not Hispanic male | 18 | 3.7 | 3.5 | 5.8 | 4.0 | 4.4 | 2.3 | 2.9 | 2.1 | 6.0 | 1.7 | 5.8 | 1.9 |
